# Regulation of Cellular and Cancer Stem Cell-Related Putative Gene Expression of Parental and CD44^+^CD24^−^ Sorted MDA-MB-231 Cells by Cisplatin

**DOI:** 10.3390/ph14050391

**Published:** 2021-04-21

**Authors:** May Zie Koh, Wan Yong Ho, Swee Keong Yeap, Norlaily Mohd Ali, Lily Boo, Noorjahan Banu Alitheen

**Affiliations:** 1 Faculty of Sciences and Engineering, University of Nottingham Malaysia, Semenyih 43500, Malaysia; mayzie.koh@gmail.com; 2 China-ASEAN College of Marine Sciences, Xiamen University Malaysia, Sepang 43900, Malaysia; 3 Faculty of Medicine and Health Sciences, Universiti Tunku Abdul Rahman, Cheras 43000, Malaysia; norlailyma@gmail.com (N.M.A.); boolily83@gmail.com (L.B.); 4 Faculty of Biotechnology and Biomolecular Sciences, Universiti Putra Malaysia, Serdang 43400, Malaysia; noorjahan@upm.edu.my

**Keywords:** triple-negative breast cancer, cisplatin resistance, cancer stem cells, Hedgehog pathway, angiogenesis

## Abstract

Triple-negative breast cancer (TNBC) is an aggressive breast cancer subtype that promotes a higher risk of metastasis and cancer reoccurrence. Cisplatin is one of the potential anticancer drugs for treating TNBC. However, the occurrence of cisplatin resistance still remains one of the challenges in fully eradicating TNBC. The presence of cancer stem cells (CSCs) has been proposed as one of the factors contributing to the development of cisplatin resistance. In this study, we aimed to characterize the cellular properties and reveal the corresponding putative target genes involved in cisplatin resistance associated with CSCs using the TNBC cell line (MDA-MB-231). CSC-like cells were isolated from parental cells and the therapeutic effect of cisplatin on CSC-like cells was compared to that of the parental cells via cell characterization bioassays. A PCR array was then conducted to study the expression of cellular mRNA for each subpopulation. As compared to treated parental cells, treated CSCs displayed lower events of late apoptosis/necrosis and G2/M phase cell arrest, with higher mammosphere formation capacity. Furthermore, a distinct set of putative target genes correlated to the Hedgehog pathway and angiogenesis were dysregulated solely in CSC-like cells after cisplatin treatment, which were closely related to the regulation of chemoresistance and self-renewability in breast cancer. In summary, both cellular and gene expression studies suggest the attenuated cytotoxicity of cisplatin in CSC-like cells as compared to parental cells. Understanding the role of dysregulated putative target genes induced by cisplatin in CSCs may aid in the potential development of therapeutic targets for cisplatin-resistant breast cancer.

## 1. Introduction

Triple-negative breast cancer (TNBC) is a breast cancer subtype defined by its lack of an estrogen receptor (ER) and progesterone receptor (PR), and the absence of amplification of human epidermal growth factor receptors 2 (HER-2) [1]. Globally, TNBC accounts for approximately 10–24% of all breast cancer cases, and is more common in Asian countries [2]. In Malaysia, the incidence of TNBC has been reported to range from 12.3% to 17.6% of the total breast cancer cases [3,4,5]. TNBC displays more aggressive behavior than other breast cancer subtypes, and is strongly associated with the recurrence of metastatic breast cancer, and metastasis is a major cause of patient morbidity and mortality [6,7].

Due to the absence of all three receptors, treatment for TNBC patients is highly challenging as no defined targets are available. Although not currently considered a standard drug for TNBC, clinical trials using mono-treatment with cisplatin and combinations with other drugs have been reported, in which cisplatin was used in neoadjuvant treatment [8,9] as well as a treatment for metastatic TNBC [10]. Cisplatin is capable of targeting the DNA repair complex of TNBC by forming DNA adduct to inhibit DNA replication, leading to cell-cycle arrest and apoptosis [11,12]. In addition, cisplatin is also able to dissociate p63 and p73, the transcription factors that are co-expressed exclusively in TNBC. The binding of these proteins suppressed pro-apoptotic activities in TNBC. Therefore, dissociation of the proteins would activate p73-dependent transcription of pro-apoptotic Bcl-2 family members, leading to apoptosis [13]. Thus, treatment regimes involving cisplatin have shown promising results for most TNBC patients [14,15,16], renewing the interest in the use of cisplatin to treat TNBC [17]. However, the efficacy of cisplatin on TNBC is still not optimal due to the frequent occurrence of drug resistance [18,19,20,21], which remains the major obstacle for the effective clinical application of cisplatin.

On the other hand, many studies have demonstrated the presence of cancer stem cells (CSCs) as one of the leading causes of drug resistance in cancer patients. A previous study demonstrated CSCs to be a contributor to cisplatin resistance developed in basal-like breast cancer [22]. Thus, the presence of CSCs in TNBC could be one of the factors for the development of cisplatin resistance. In fact, previous studies demonstrated overexpression of apoptosis-related proteins in CSCs, including survivin [23,24], cellular inhibitors of apoptosis, inhibitors of apoptosis protein (IAP) [25] and cell cycle regulatory components that inhibit apoptosis, leading to higher chemoresistance [26]. Apart from displaying higher chemoresistance, CSCs are known to be highly self-renewable. For example, Moreover, CSCs were found to be more likely to metastasize to secondary sites, and later generate a secondary tumor via their self-renewability [27].

Over the past decades, researchers have demonstrated that low CD24 expression and high CD44 (CD24^−^/CD44^+^) is the standard biomarker for breast CSCs. These biomarkers were first discovered by Al-Hajj et al. (2003), showing that as low as 100 cells expressing CD24^−^/CD44^+^ were able to generate tumors in mice, whereas thousands of non CD24^−^/CD44^+^ cells failed to do so [28]. Following this discovery, many have utilized this CSC phenotype as a biomarker for breast CSCs isolation [29,30,31]. Indeed, Ghebeh et al. (2013) compared the stem-cell properties of CSCs isolated from different biomarkers and concluded that the CD24^−/low^/CD44^+^ phenotype was the predominant biomarker for breast CSCs among other biomarkers [32]. Furthermore, enriched aldehyde dehydrogenase 1 (ALDH1) activity is one of the features of breast CSCs previously discovered by Ginestier et al. (2007), and elevation of ALDH1A1 activity was later found to be correlated with tumorigenesis and drug resistance in breast cancer [33,34,35].

Therefore, the present study aims to investigate the contribution of the CSC subpopulation to cisplatin resistance in TNBC and the putative target genes that are dysregulated in CSCs as compared to parental cells in response to cisplatin treatment. Likewise, signaling pathways that are associated with these putative target genes can also be identified to support more effective treatment strategies. To address the research questions, CSC-like cells isolated from the TNBC cancer cell line, MDA-MB-231, which is one of the most commonly utilized TNBC cell lines for CSC studies, were used in this study to investigate the cisplatin-induced alteration of cellular and putative target gene expression characteristics.

We report that CSC-like cells are more resistant to cisplatin treatment, supported by lower late apoptosis/necrosis, higher expression of an CSC-like phenotype and self-renewability as compared to its parental counterpart after cisplatin treatment. Furthermore, dysregulation of putative target genes such as Smoothened (SMO), Glycogen Synthase Kinase 3 Beta (GSK3β), Notch Receptor 2 (NOTCH2), Delta-like protein 1 (DLL1) and WNT1, which were correlated with the Hedgehog signaling pathway and angiogenesis in CSC-like cells, may attenuate cisplatin cytotoxicity in TNBC, and this discovery provides a potential approach to improve the efficacy of cisplatin in TNBC therapy.

## 2. Results

### 2.1. IC_50_ Value of Cisplatin on Parental MDA-MB-231

**Figure 1:**
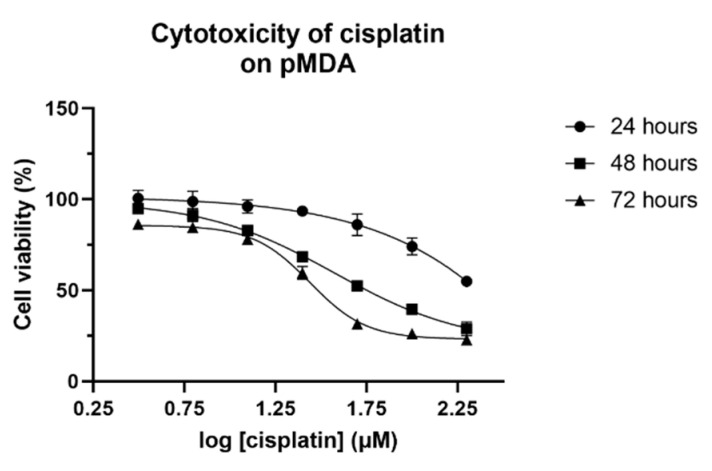
Cell viability of pMDA after 24, 48 and 72 h of cisplatin treatment. The values are represented as means ± SD for triplicates of independent experiments.

Cisplatin displayed its cytotoxic effect in a dose-dependent manner in parental MDA-MB-231 cells (pMDA). The IC_50_ values of cisplatin were >200 µM, 56.27 ± 2.59 µM (95% confidence interval (CI) 53.7–58.88; r^2^ = 0.99) and 30.51 ± 2.60 µM (95% CI 27.54–32.36; r^2^ = 0.99) for 24, 48 and 72 h, respectively (Figure 1), as determined by non-linear regression analysis. The shortest time point at which cells achieved a stable cytotoxicity effect was selected to acquire cisplatin-treated subpopulations. The acquired IC_50_ value of pMDA was reduced drastically by >3.5-fold at 48 h as compared to 24 h, but to a much lower extent (1.9-fold) at 72 h as compared to 48 h, indicating 48 h was the shortest tested time point to achieve a stable cisplatin cytotoxic effect. Therefore, the IC_50_ value of cisplatin treatment at 48 h (56 µM) was selected to treat both pMDA and the sorted CD24^−^/CD44^+^ CSC-like subpopulation (sMDA) to study the effect of cisplatin on sMDA.

### 2.2. Sorted MDA-MB-231 Retains Higher Cancer Stem Cell Phenotype Than Parental MDA-MB-231 after Cisplatin Treatment

**Figure 2:**
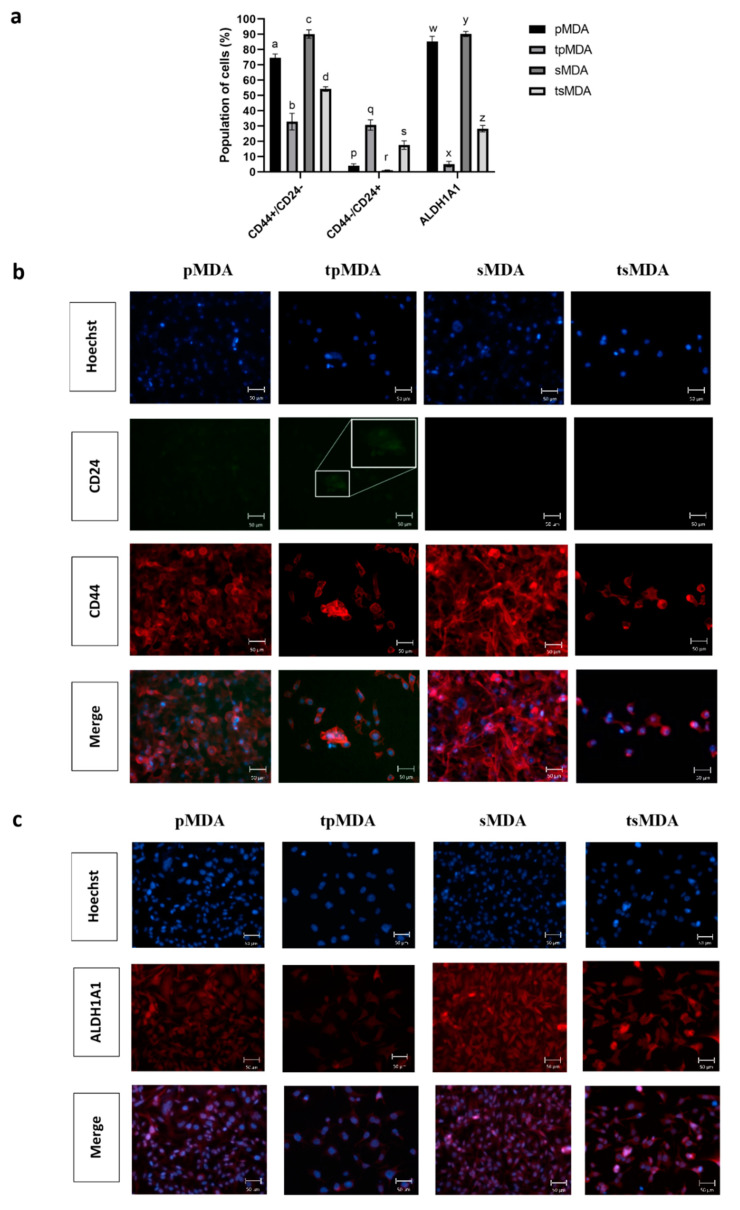
Breast cancer stem cell markers detected using flow cytometry and fluorescent microscopy. (**a**) Fold change of the cellular population expressing CD24^−^/CD44^+^, CD24^+^ and ALDH1A1 on the control pMDA or sMDA vs cisplatin-treated tpMDA and tsMDA, respectively. Data are presented as means ± SD for triplicates of independent experiments, with different letters showing significant differences where *p* < 0.05. (**b**) Immunofluorescence imaging of control and treated pMDA and sMDA at 20× objective magnification, stained by anti-CD24-FITC (green), anti-CD44-Alexa Fluor 647 (red) and Hoechst stain (blue); scale bar: 50 μm, with a zoomed inset presenting CD24 detected in tpMDA. (**c**) Control and treated pMDA and sMDA at 20× objective magnification, stained by anti-ALDH1A1-APC (red) and Hoechst stain (blue), under the respective filters, along with a merged image, scale bar: 50 μm.

Cisplatin decreased the expression of CSCs markers CD24^−^/CD44^+^ and ALDH1A1 on pMDA and sMDA significantly compared to their respective controls, but to a lower extent in sMDA (Figure 2a). Under the same treatment conditions, cisplatin reduced a higher percentage of CSC-like cells (CD24^−^/CD44^+^) in cisplatin-treated pMDA (tpMDA) than the CSC-like cells in cisplatin-treated sMDA (tsMDA), as compared to their respective controls (Figure 2a). On the other hand, cisplatin treatment led to a higher percentage of CD24^+^/CD44^−^ cells in pMDA than sMDA (Figure 2a). More significantly, cisplatin treatment significantly reduced the population of cells expressing ALDH1A1 in tpMDA, compared to tsMDA (Figure 2a).

In accordance with the results obtained from flow cytometry analysis, the immunofluorescent microscope imaging showed a higher number of CD24^−^/CD44^+^ and ALDH1A1-expressing cells in tsMDA than in tpMDA, as visualized by the higher intensity of the red signal (Figure 2b,c).

On the other hand, CD24 expression was significantly increased in tpMDA (Figure 2b), complemented by the observed faint CD24 green signal (indicated by a white circle in the Figure) as compared to pMDA (Figure 2b). Although the increment of the CD24 green signal in tsMDA relative to sMDA was not observable, tsMDA displayed relatively lower expression of CD24 markers and equally visible CD44 expression, and acquired more CSC features as compared to tpMDA.

Considering together the quantitative and qualitative results obtained for both CSC biomarkers, the sorted subpopulation retained a higher percentage of CSC-like cells after cisplatin treatment as compared to its parental cells.

### 2.3. Sorted MDA-MB-231 Is More Resistant to Anticancer Drugs

**Figure 3:**
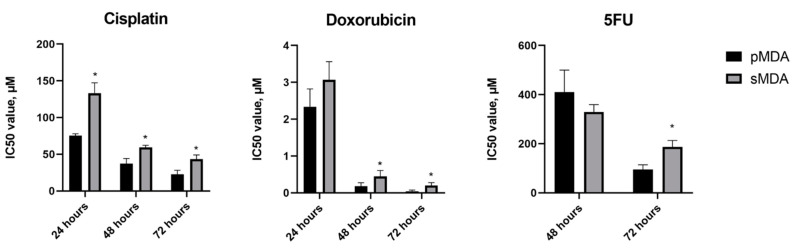
The IC_50_ values (μM) of chemotherapeutic drugs, including cisplatin, doxorubicin and 5FU in pMDA and sMDA for 24, 48 and 72 h, determined via the Real-Time Glo assay. All data were expressed as means ± SD for triplicates of independent experiments. * represents significant differences (*p* < 0.05) as compared to pMDA.

sMDA demonstrated higher resistance to all three drugs as compared to pMDA (Figure 3). The IC_50_ values of cisplatin, doxorubicin and 5FU were determined by means of non-linear regression analysis. The IC_50_ values of cisplatin for pMDA were 75.31 ± 2.63 µM (95% CI 72.44–77.62; r^2^ = 0.95), 37.19 ± 6.98 µM (95% CI 29.51–43.16; r^2^ = 0.96) and 22.50 ± 5.70 µM (95% CI 17.78–28.84; r^2^ = 0.96), whereas for sMDA they were 133.30 ± 13.90 µM (95% CI 120.20–147.90; r^2^ = 0.80), 59.38 ± 2.82 µM (95% CI 56.23–61.66; r^2^ = 0.92) and 43.49 ± 5.64 µM (95% CI 36.98–46.77; r^2^ = 0.90) for 24, 48 and 72 h, respectively. Moreover, IC_50_ values of doxorubicin for pMDA were 2.34 ± 0.48 µM (95% CI 1.95–2.88; r^2^ = 0.90), 0.18 ± 0.10 µM (95% CI 0.11–0.29; r^2^ = 0.91) and 0.04 ± 0.04 µM (95% CI 0.01–0.08; r^2^ = 0.89), whereas for sMDA they were 3.07 ± 0.49 µM (95% CI 2.57–3.55; r^2^ = 0.78), 0.44 ± 0.17 µM (95% CI 0.26–0.58; r^2^ = 0.84) and 0.20 ± 0.08 µM (95% CI 0.11–0.26; r^2^ = 0.86) for 24, 48 and 72 h, respectively. The determined IC_50_ values across 24, 48 and 72 h for cisplatin ranged between 1.6- to 1.9-fold higher for respective treatments in sMDA as compared to pMDA, and were 1.3-fold, 2.5-fold and 5.0-fold higher in sMDA than that of pMDA for 24, 48 and 72 h, respectively, for doxorubicin. With almost equally high tolerance to 5FU, both pMDA and sMDA displayed IC_50_ values beyond 500 μM at 24 h (result not shown) and insignificantly different IC_50_ values at 48 h of 5FU treatment. However, similar to the trend of the IC_50_ values acquired from the other two chemotherapeutic drugs, sMDA, with an identified IC_50_ value of 187.40 ± 25.83 µM (95% CI 162.20–213.80; r^2^ = 0.91), showed a 2.0-fold higher IC_50_ value than that of pMDA, which displayed 95.37 ± 18.75 µM (95% CI 74.13–109.70; r^2^ = 0.92) at 72 h of 5FU treatment.

### 2.4. Sorted MDA-MB-231 Is More Resistant to Cisplatin-Induced Apoptosis

The reduction of cell viability after cisplatin treatment is mainly due to the induction of apoptosis. In this study, the effects of cisplatin on apoptosis and cell cycle distribution in pMDA and sMDA were evaluated via the FITC Annexin V/PI Apoptosis Detection Kit and the BD Cycletest plus DNA kit, respectively.

**Figure 4:**
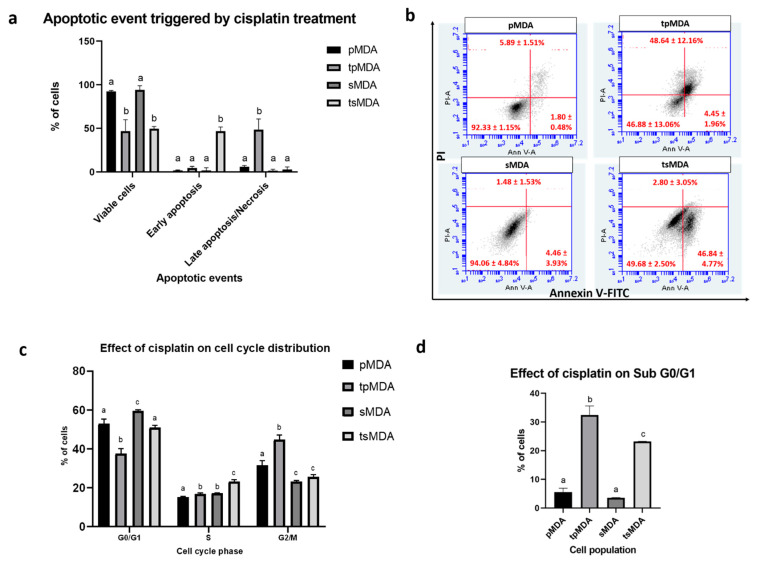
Comparison of apoptotic events and cell cycle distribution of pMDA and sMDA after cisplatin treatment. (**a**) Apoptotic event triggered by cisplatin treatment. (**b**) Representative AnnexinV/PI flow cytometry data for pMDA, tpMDA, sMDA and tsMDA. (**c**) Effect of cisplatin treatment on cell cycle distribution of pMDA and sMDA exclusively on the G0/G1 phase, S phase and G2/M phase and (**d**) solely on the sub-G0/G1 phase. Data are presented as means ± SD for triplicates of independent experiments, with different letters showing significant differences where *p* < 0.05.

Cisplatin exerted a different degree of apoptosis in pMDA and sMDA, demonstrated by 42.52% significantly higher (*p* = 0.0001) early apoptosis, with 45.84% significantly lower (*p* = 0.0032) late apoptosis/necrosis in tsMDA as compared to tpMDA (Figure 4a,b). Moreover, the effects of cisplatin exclusively on G0/G1, S and G2/M phases for treated subpopulations were evaluated by excluding the sub-G0/G1 phase to precisely identify the interference of cisplatin treatment on these phases (Figure 4c). As compared to their respective controls, cisplatin triggered a higher and more significant cell increment in the S phase in tsMDA (increased by 5.98%; *p* < 0.0001) than that of tpMDA (increased by 1.53%; *p* = 0.043), whereas tpMDA alone displayed a significant (*p* < 0.0001) cell increment in the G2/M phase, by 13.12% as compared to pMDA. Furthermore, as compared to their respective controls, the increases in sub-G0/G1 content induced by cisplatin treatment were 7.2% significantly lower (*p* = 0.0008) in tsMDA (increased by 19.63%) than that of tpMDA (increased by 26.83%) (Figure 4d). These data showed that cisplatin induced a lower increase in sub-G0/G1 content in tsMDA, and at the same time induced tsMDA to be arrested at the S phase, whereas tpMDA was arrested more significantly at the G2/M phase.

### 2.5. Sorted MDA-MB-231 Retains Remarkably Higher Self-Renewability after Treatment

**Figure 5:**
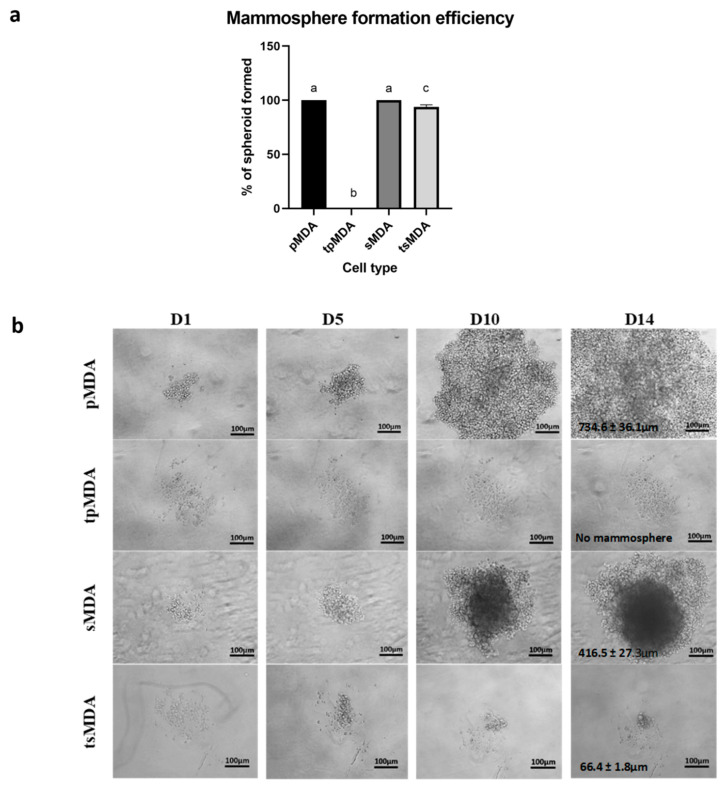
Percentage and sizes of generated mammospheres in each subpopulation. (**a**) The number of generated mammospheres was assessed after 14 days. (**b**) Images of generated mammospheres were captured using phase-contrast microscopy at 10× objective magnification on day 14; scale bar: 100 μm. Size of the mammospheres and images are representative of three biological replicates. Data were presented as means ± SD for triplicates of independent experiments, with different letters showing significant differences where *p* < 0.05.

Both pMDA and sMDA controls generated 100% of mammospheres before treatment, with larger but less compact mammospheres observed in pMDA (734.6 ± 36.1 µm) and smaller but more compact mammospheres observed in sMDA (416.5 ± 27.3 µm) after 14 days in culture. Although both pMDA and sMDA possessed similarly high self-renewability before cisplatin treatment, only tsMDA retained its self-renewability despite the significant reduction, whereas tpMDA lost its self-renewability completely after cisplatin treatment. As the result, tsMDA displayed approximately 90.97% significantly higher (*p* < 0.0001) self-renewability than that of tpMDA (Figure 5a). Conversely, tsMDA then generated larger mammospheres (68.4 ± 1.8 µm), whereas tpMDA failed to generate mammospheres after cisplatin treatment (Figure 5b). These data suggested that cisplatin reduced the self-renewability of sMDA to a remarkably lower extent as compared to pMDA, which completely lost its self-renewability after cisplatin treatment.

### 2.6. Dysregulation of Cancer Stem Cell-Correlated Putative Target Genes in MDA-MB-231 Parental and Sorted MDA-MB-231 Induced by Cisplatin Treatment

The RT^2^ profiler PCR array, a qPCR-based method with the features of a mini-array, was used to study cellular gene expression, particularly on functionally correlated putative target genes in CSCs before and after cisplatin treatment in order to decipher the alteration of putative gene profiles in pMDA and sMDA subpopulations.

**Figure 6:**
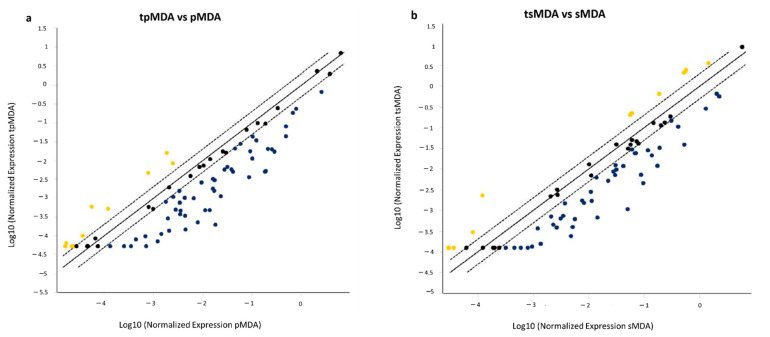
Scattered plots of CSC-related putative gene expression. CSC-related putative gene expression profiles of (**a**) tpMDA and (**b**) tsMDA as compared to their respective controls. Upregulated and downregulated putative genes are represented by yellow and blue dots respectively.

Cisplatin induced more genes to be downregulated by at least two-fold (40% and 55% of genes in tpMDA and tsMDA, respectively), with fewer upregulated genes (2% and 23% of genes in tpMDA and tsMDA, respectively) in both treated subpopulations as compared to their respective controls (Figure 6).

### 2.7. Dysregulated Cancer Stem Cell-Correlated Putative Target Genes and Corresponding Signaling Pathways Induced by Cisplatin Treatment

**Figure 7:**
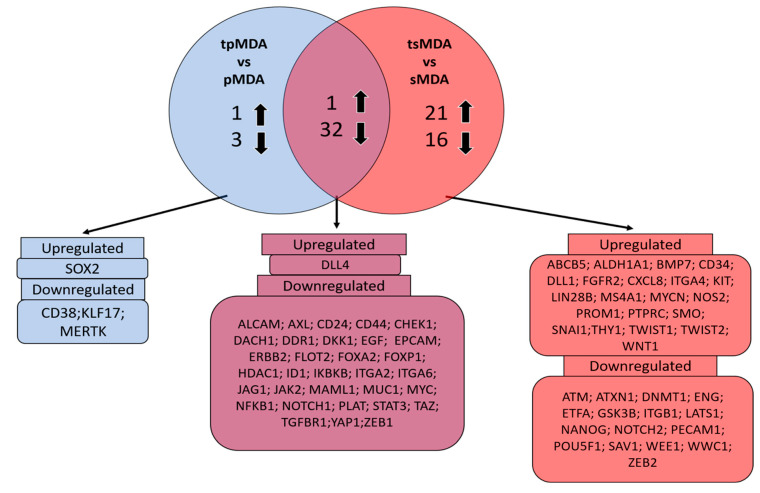
Venn diagram of cisplatin-induced dysregulation of putative target genes in pMDA and sMDA. Numbers of significantly dysregulated CSC-correlated putative target genes (≥2-fold, *p* < 0.05) in pMDA and sMDA subpopulations after cisplatin treatment, with uniquely and commonly expressed putative target genes in tpMDA vs pMDA control and tsMDA vs sMDA control.

**Figure 8:**
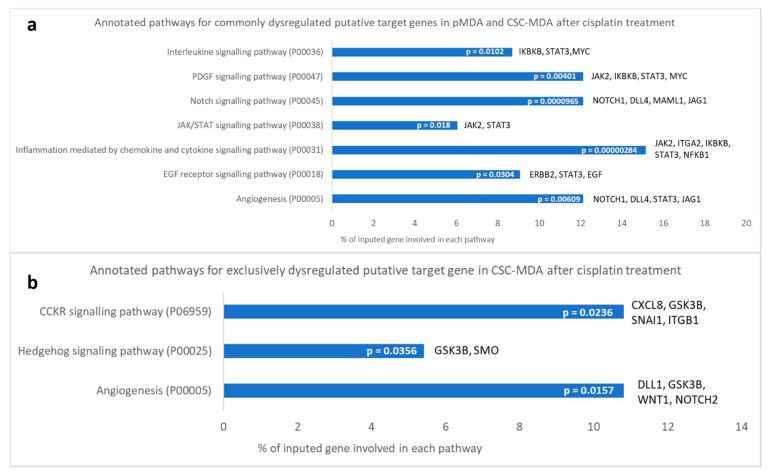
PANTHER pathway analysis. Annotated signaling pathways correlated to (**a**) commonly dysregulated CSC-correlated putative target genes (≥2-fold, *p* < 0.05) in pMDA and sMDA after cisplatin treatment, and (**b**) exclusively dysregulated CSC-correlated putative target genes (≥2-fold, *p* < 0.05) in sMDA after cisplatin treatment. The corresponding adjusted Fisher’s exact *p*-values (*p* < 0.05) are presented for each pathway.

Based on the determined dysregulated putative gene profiles in respective subpopulations, signaling pathways correlated with these dysregulated putative target genes were annotated via the PANTHER overrepresentation test (Figure 7 and Figure 8).

Cisplatin-induced, significantly dysregulated putative target genes (≥2-fold, *p* < 0.05) in pMDA and sMDA (relative to their respective controls) were compared to identify the commonly and distinctively dysregulated putative gene profiles between pMDA and sMDA (Figure 7). There were 33 commonly dysregulated putative target genes between pMDA and sMDA after cisplatin treatment—up to 32 putative target genes were downregulated, with only 1 upregulated putative target gene detected (Figure 7). However, the only upregulated putative target gene (DLL4) displayed a negligible difference of only 0.1-fold between the increment in sMDA (2.85-fold) and pMDA (2.74-fold) as compared to their respective controls (Supplementary Table S1). A number of these commonly dysregulated putative target genes were significantly annotated to seven signaling pathways (Figure 8a). Among these pathways, more than 10% of these putative target genes were involved in ‘platelet-derived growth factor (PDGF)’, ‘Notch’ and ‘angiogenesis’ pathways, which are correlated with breast cancer progression. Moreover, there were four significantly dysregulated putative target genes solely in the pMDA subpopulation after cisplatin treatment, comprising one upregulated and three downregulated putative target genes (Figure 8 and Supplementary Table S2). No significant pathway was annotated based on these dysregulated genes in pMDA after cisplatin treatment (result not shown). On the other hand, there were 37 significantly dysregulated putative target genes solely in the sMDA subpopulation after cisplatin treatment, comprising 21 upregulated and 16 downregulated putative target genes (Figure 7 and Supplementary Table S2). Eight of these exclusively dysregulated putative target genes in tsMDA were significantly annotated to three signaling pathways—‘Hh’, ‘cholecystokinin receptor (CCKR)’ and ‘angiogenesis’ pathways (Figure 8b).

## 3. Discussion

The present study described the effect of cisplatin on CSC-like subpopulations in comparison to its parental cells in the TNBC cell line, MDA-MB-231. This study focused on the effect of cisplatin on the cellular characteristics of both sorted CSC-like (sMDA) and parental (pMDA) MDA-MB-231 subpopulations. Furthermore, the differential expression of the CSC-related putative target genes of the model was also evaluated. Therefore, this study was intended to provide a better understanding of the correlation of CSC-like subpopulation to the efficacy of cisplatin treatment in TNBC.

Cisplatin is able to activate apoptosis in cancer cells via DNA damage induced by formation of DNA adducts [36,37]. In this study, tpMDA displayed significant apoptotic events, with majority of cells trapped in late apoptosis/necrosis (Figure 4a,b). In complement to the apoptotic event, tpMDA showed a significant increase in sub-G0/G1 events, which represent fragmented DNA (Figure 4d), one of the hallmarks of cell apoptosis [38]. In addition, tpMDA was arrested in the S and G2/M phases, with a higher rate and significance observed in the G2/M phase (Figure 4c)—prolonged G2/M arrest could also lead to apoptosis [39,40,41]. However, accumulating evidence suggests that CSCs in the breast are more resistant to the induction of apoptosis by conventional chemotherapy [42,43,44,45,46]. sMDA displayed generally higher IC_50_ values than pMDA, showing higher chemoresistance not only to cisplatin, but to all of the tested anticancer drugs (Figure 3). Cisplatin activated a milder degree of apoptosis in sMDA as the majority of the tsMDA were still undergoing early apoptosis, whereas most of the tpMDA had already progressed to late apoptosis/necrosis after exposure to cisplatin treatment (Figure 4a,b). A significant increment in sub G0/G1 events was also observed in tsMDA but the increment was lower than that of tpMDA as compared to the pMDA controls (Figure 4d). Furthermore, tsMDA displayed a higher number of cells arrested in the S phase than that of tpMDA (Figure 4c), suggesting the possibility of a greater DNA repair mechanism in the sorted breast CSC-like subpopulation, in accordance with lung CSCs that demonstrated enhanced ability to implement DNA repair by arresting cells in the DNA-replicating S phase after being treated by cisplatin [47]. In fact, a previous study showed that breast cancer MCF-7 cells were arrested at the G1/S phase when sub-lethal cisplatin concentrations were used, whereas higher doses and higher cisplatin concentrations induced G2/M arrest, preceding cell death [48]. Thus, cisplatin may have exerted a high-dose lethal effect on tpMDA, which significantly arrested cells in the G2/M phase, but a sub-lethal effect on tsMDA, resulting in cell arrest in the S phase, although same cisplatin treatment condition was applied. Thus, the cell arrest in the S phase observed for tsMDA in this study could be a protective effect against cisplatin treatment.

Drug resistance characteristics were correlated to the higher expression of ALDH [34] and CD24^−^/CD44^+^ [49,50] which are the key markers in CSCs. A previous study has shown that inhibition of ALDH activity via all-trans-retinoic acid (ATRA) or specific ALDH inhibitor diethylaminobenzaldehyde (DEAB) in MDA sensitized the initially chemo- and radio-resistant ALDH^+^ breast cancer cells to the applied treatment [42]. Moreover, knockdown of ALDH1A1 in a TNBC cell line (MDA-MB-468) sensitized breast cancer cells to paclitaxel, doxorubicin and radiation therapy [51]. Not only that, previous studies also reported that CD24^−^/CD44^+^-expressing breast CSCs in MDA were more resistant to doxorubicin than that of non-CSCs (CD24^+^/CD44^+^) [50]. In this study, cisplatin inhibited the expression of breast CSC biomarkers (CD24^−^/CD44^+^ and ALDH1A1) in tpMDA (Figure 2) at a higher extent than the sorted MDA. On the other hand, the population of cells expressing CD24 was increased in both cisplatin-treated pMDA and sMDA. CD24 was found to be positively correlated to cell differentiation [52,53,54]. Cisplatin, which increased CD24 expression, is suggested to induce stem-to-progenitor differentiation in tpMDA and tsMDA, and this cisplatin-triggered cell differentiation was previously reported in breast cancer [49]. However, the results have shown that cisplatin more specifically targeted progenitor cells, rather than the stem breast cancer cells, with the reduction of the population of CD24^−^/CD44^+^ and ALDH1A1 MDA-MB-231 cells being at higher folds in tpMDA, compared to tsMDA (Figure 2). These data suggest that tsMDA retained a higher stem cell phenotype and was differentiated to a lower extent as compared to tpMDA, which contributed to greater resistance to the anticancer drugs as compared to pMDA, similarly to the previous findings [34,49,50]. Moreover, cisplatin demonstrated a remarkable inhibitory effect on the self-renewability of tpMDA, with the fully self-renewable pMDA being unable to generate mammospheres completely after being treated with cisplatin (Figure 5). This result is consistent with a previous study that demonstrated the capability of 100–200 self-renewable CD24^−^/CD44^+^-expressing breast cancer cells to initiate tumor growth in non-obese diabetic/severe combined immunodeficient (NOD/SCID) immunocompromised mice, whereas 20,000 of CD24^+^/CD44^+^-expressing cells failed to do so [28]. The higher stem cell phenotype of sMDA and tsMDA contributed to the higher self-renewable characteristic, which was shown by the ability to generate solid mammospheres, compared to pMDA and tpMDA, suggesting that the sorted subpopulation is more tumorigenic as compared to its parental cells (Figure 5). Taken together, cisplatin induced lower suppression of the stem cell phenotype, a lower apoptotic effect, less fragmented DNA, cell cycle arrest in the S phase and lower suppression of self-renewability in tsMDA as compared to its parental cells. This suggests that the sorted subpopulation possessed features of tumorigenic breast CSCs, which may contribute to attenuation of cisplatin efficacy in TNBC.

To compare the differential expression of CSC-related putative target genes of pMDA and sMDA when treated with cisplatin, a PCR array was performed. Most (97%) of the significantly dysregulated putative target genes (≥2-fold, *p* < 0.05) that were common between treated subpopulations vs. their respective controls were downregulated, whereas only one gene was upregulated (Figure 7). These data suggests that cisplatin in general hindered the expression of CSC-correlated putative target genes in both sMDA and pMDA, but to a different extent. For example, approximately 60% of these commonly dysregulated putative target genes were downregulated to a lower extent in the sMDA subpopulation than the pMDA subpopulation after treatment (Figure 7 and Supplementary Table S1). The only upregulated putative target gene in both subpopulations after treatment was DLL4, with a negligible difference of 0.1-fold between the increment in both treated subpopulations as compared to their respective controls. The remaining 36% of commonly dysregulated putative target genes were downregulated to a higher extent in the sMDA subpopulation after treatment when compared to the pMDA subpopulation (Figure 7 and Supplementary Table S1). Among these commonly dysregulated putative target genes, a total of six genes were associated with ‘Notch’ and ‘angiogenesis’ signaling pathways (NOTCH1, DLL4 and Jagged1 (JAG1)), as well as the ‘PDGF signaling pathway’ (Janus Kinase 2 (JAK2), Inhibitor of Nuclear Factor Kappa B Kinase Subunit Beta (IKBKB) and MYC). These downregulated putative target genes were suppressed to a lower extent in the sMDA subpopulation compared to the pMDA subpopulation after cisplatin treatment (Figure 8a).

The lower suppression of the members of Notch signaling pathway such as NOTCH1 and JAG1 in the tsMDA subpopulation after treatment could be one of the factors that contributes to its better maintenance of the CSCs subpopulation, and the higher resistance of tsMDA than that of tpMDA (Figure 4a,b). For instance, a previous study showed that elevated Notch1, one of the receptors for the Notch pathway, was found to enhance ALDH1 levels in breast CSCs via the Notch signaling pathway [55]. Consistent with previous studies, this study shows tsMDA to have a lower reduction of NOTCH1 gene expression, displaying higher ALDH1 activity as compared to tpMDA (Figure 2c). Moreover, JAG1, a ligand for Notch receptor, was previously found to be upregulated in drug-resistant breast cancer cells, and this CSC-driven drug resistance in ER^+^ breast cancer was correlated to the interaction of JAG1 and NOTCH4 in association with the Notch signaling pathway [56]. Apart from being involving in the Notch pathway, NOTCH1 and JAG1 are also implicated in angiogenesis, which is the process of the recruitment of new blood vessels [57]. A previous study showed that Jag1 ligand overexpression promoted neovascularization and the growth of experimental tumors in mice [58]. Furthermore, a later study demonstrated the role of JAG1 as an antagonist for DLL4-mediated Notch signaling within the sprout, resulting in growth of new vessels [59]. In this study, cisplatin downregulated gene expression of JAG1 to a lower extent in the sMDA subpopulation, whereas it elevated DLL4 in both tsMDA and tpMDA (Supplementary Table S1), suggesting that cisplatin can potentially suppress angiogenesis for both subpopulations but possibly to a lower extent in sMDA. In addition, the lower suppression of MYC, a gene that correlates to the PDGF signaling pathway, in the sMDA subpopulation after treatment may be one of the causes of the higher drug resistance of tsMDA than that of tpMDA as compared to their respective controls (Figure 4a,b). For example, a previous study showed that the activation of the PDGF signaling pathway in MYC-enriched human mammary epithelial (HME) cells—which has a similar phenotype and cellular behavior to the TNBC cell line (MDA) [60]—can induce a faster and higher Ca^2+^ increase than that observed in non MYC-enriched HME cells. This increase in Ca^2+^ then triggered the higher expression of fatty acid β-oxidation (FAO) [61] and consequently enhanced drug resistance and the maintenance of stemness in breast cancer cells [62]. Furthermore, other studies have reported that breast cancer patients with elevated PDGF levels showed lower responses to chemotherapy and shorter duration of survival [63,64]. A recent study also demonstrated the correlation of an aggressive biological phenotype with the PDGF signaling pathway in TNBC by displaying significant upregulation of intracellular PDGFRα in lymph node metastases and asynchronous recurrences [65].

As a side note, Plasminogen Activator, Tissue Type (PLAT) was most downregulated putative target gene discovered among the commonly dysregulated genes for both treated subpopulations (Supplementary Table S1). A previous study demonstrated its correlation with anti-apoptosis and tumor proliferation, findings that the knockdown of PLAT restored anticancer effect of gefitinib in non-small cell lung cancer (NSCLC) [66]. In this study, PLAT putative target gene expression was highly suppressed in tsMDA and tpMDA as compared to their respective controls, but to a lower extent in tsMDA after cisplatin treatment, suggesting anti-proliferate activity and the induction of apoptosis in both treated subpopulations, but less effectively in tsMDA, supported by the fewer events of late apoptosis/necrosis observed in tsMDA as compared to tpMDA after cisplatin treatment (Figure 4a,b).

Notably, of all the significantly dysregulated putative target genes in tsMDA, approximately 53% of these genes were dysregulated exclusively in the sMDA subpopulation after cisplatin treatment (Figure 7). Among these putative target genes, five are correlated to the ‘Hh signaling pathway’ (SMO and GSK3β) and ‘angiogenesis’ (NOTCH2, DLL1, WNT1 and GSK3β) (Figure 8b). The Hh-pathway-correlated-gene expression of SMO was upregulated, whereas GSK3β was downregulated in tsMDA as compared to its control (Supplementary Table S2). As the activation of the Hh signaling pathway, mediated by SMO, is crucial for the maintenance of CSCs and the self-renewability of breast cancer cells [67], whereas GSK3β is positively correlated to apoptosis in the TNBC cell line (MDA) [68], the upregulation of SMO and the downregulation of GSK3β exclusively in tsMDA may contribute to higher cisplatin resistance and self-renewal ability in tsMDA, compared to tpMDA (Figure 4a,b and Figure 5).

Moreover, putative target genes involved in angiogenesis, including the upregulated DLL1 and WNT1 and downregulated GSK3β and NOTCH2, might enhance the aggressiveness of tsMDA. Furthermore, inhibition of WNT1 was previously reported to enhance apoptosis [69] and suppress self-renewability in breast CSCs in vitro [70]. On the other hand, NOTCH2, encoded for the receptor of Notch signaling pathway, was reported to suppress tumor growth in human breast cancer xenografts [71]. Notably, although activation of Notch2 induced higher vascularization, the vessels were smaller and comprised a more immature network as compared with Notch4-activated signaling, suggesting its suppressive role in the angiogenesis of breast cancer [71]. In reference to these previous findings, the upregulation of DLL1 and WNT1 exclusively in tsMDA observed in this study may play a part in inducing higher anti-apoptotic and self-renewal abilities in tsMDA compared to tpMDA (Figure 4a,b and Figure 5), whereas the downregulation of NOTCH2 may suggest that tsMDA possessed greater potential to initiate angiogenesis as compared to tpMDA.

In agreement with the observed cellular characteristics of cisplatin-treated subpopulations, putative Notch and PDGF pathways, which are positively correlated to breast cancer progression, were hindered to a lower extent in tsMDA after treatment. Furthermore, putative Hh and angiogenesis pathways that were exclusively enriched in sMDA after treatment could be the core putative pathways contributing to the attenuation of cisplatin effects, including maintaining self-renewability, inhibiting apoptosis and inducing more aggressive behavior in the sorted subpopulation as compared to the parental subpopulation after treatment. Therefore, putative target gene expression analysis supported the observed cellular characteristics of tsMDA as being more tumorigenic and resistant to cisplatin treatment than its parental counterpart. This indicates that the sorted CSC-like subpopulations played a vital role in the acquirement of cisplatin resistance in TNBC.

## 4. Materials and Methods

### 4.1. Cell Culture for Triple-Negative Breast Cancer Cells

The human TNBC cell line MDA-MB-231 was purchased from ATCC (Manassas, VA, USA). MDA-MB-231 was cultured under adherent culture conditions, sustained by RPMI-1640 (Sigma-Aldrich, St. Louis, MO, USA), supplemented with 10% fetal bovine serum (FBS) (Gibco, Gaithersburg, MD, USA). Cancer cells were cultured at 37 °C in a 5% CO_2_ humidified atmosphere and were sub-cultured upon reaching 70% confluency.

### 4.2. Isolation of Triple-Negative Breast Cancer Stem Cells via Magnetic-Activated Column Sorting

Isolation of the CSC-like subpopulation was conducted via magnetic-activated cell sorting (MACS) separation, which involved a MACS Column and MACS separator (Miltenyi Biotec, Bergisch Gladbach, Germany). The parental MDA-MB-231 cells (pMDA) were filtered using a 70-µm cell strainer (BD, Franklin Lakes, NJ, USA) to obtain single cell suspension prior to cell sorting. MACS separation was conducted according to the protocol provided by manufacturer. Briefly, the 24- subpopulation was first isolated via CD24 MicroBead kit (Miltenyi Biotec, Bergisch Gladbach, Germany) by positive depletion of CD24-expressing cells, followed by positive selection of CD44-expressing cells using anti-CD44-PE (Biolegend, San Diego, CA, USA) and anti-PE MicroBead (Miltenyi Biotec, Bergisch Gladbach, Germany). This resulted in the acquisition of the sorted CD24^−^/CD44^+^ CSC-like subpopulation (sMDA).

### 4.3. Assessment for the Cytotoxicity of Cisplatin

An MTT assay (Sigma-Aldrich, St. Louis, MO, USA) was conducted to determine the half maximal inhibitory concentration (IC_50_ value) of cisplatin (Sigma-Aldrich, St. Louis, MO, USA). The pMDA cells were seeded at a cell density of 8000 cells/well in a flat-bottom 96-well plate (BD, Franklin Lakes, NJ, USA) and incubated at 37 °C in 5% CO_2_ for 24 h to achieve 70% confluency. Seeded cells were treated with a range of cisplatin from 0–200 µM. The cisplatin treated plate was incubated for 24, 48 and 72 h, followed by the addition of 5 mg/mL of MTT solution (Sigma-Aldrich, St. Louis, MO, USA). The insoluble formazan crystal formed was observed and solubilized in dimethyl sulfoxide (DMSO) (Fisher Scientific, USA) before absorbance readings were taken using a microplate reader (µ Quant Biotek Instruments, Winooski, VT, USA) at a wavelength of 570 nm. The shortest time point with stabilized drug cytotoxicity was selected for the acquisition of cisplatin-treated triple-negative breast cancer subpopulations.

### 4.4. Acquirement of Cisplatin-Treated Triple-Negative Breast Cancer Subpopulations

A seeding density of 2 × 10^5^ of cells from pMDA and MACS-sorted sMDA were seeded in a 6-well plate (BD, Franklin Lakes, NJ, USA) for 24 h until the adhered cancer cells reached 70% confluency. The confluent cells were then treated with the identified IC_50_ value of 56 µM cisplatin (Sigma-Aldrich, St. Louis, MO, USA) for 48 h to acquire the surviving subpopulation after cisplatin treatment. As a longer culturing duration can induce a higher rate of cell differentiation, it is crucial to select the shortest time point with a stabilized cisplatin cytotoxic effect, which was the acquired IC_50_ value of cisplatin at 48 h, to acquire cisplatin-treated subpopulations. This can ensure the best maintenance of the cell stemness of the MACS-sorted untreated sMDA after a total of 72 h of culture duration (including 24 h of incubation to allow adhered cells to become confluent, followed by 48 h of incubation time, similar to the treatment duration for cisplatin-treated subpopulations), prior being harvested and investigated. Therefore, a total of four MDA subpopulations as listed below were acquired and investigated by means of bioassays, as outlined in the following Sections.

- pMDA control
- Treated pMDA (tpMDA)
- sMDA control
- Treated sMDA (tsMDA)

### 4.5. Immuno-Phenotyping for Expression of Breast Cancer Stem Cell Surface Markers CD44/CD24 via Flow Cytometry

Anti-CD44 and anti-CD24 flow-cytometry was conducted to quantify CSC-like cells. Cancer cells were harvested and counted to prepare the appropriate number of cells for flow analysis. A total of 1 × 10^6^ cells/mL of the single-cell suspension were suspended in 100 µL of phosphate-buffered saline (PBS), followed by double staining with a 1:40 dilution of anti-CD24-FITC (Biolegend, San Diego, CA, USA) and anti-CD44-PE ((Biolegend, San Diego, CA, USA) in PBS. The reaction was incubated at 4 °C for at least 1 h in the dark. The cells were then washed with PBS to remove unbound dyes. The stained cell pellet was resuspended in 500 µL of fresh PBS, then subjected to a BD Accuri C6 flow cytometer and analyzed using BD Accuri C6 software (BD, Franklin Lakes, NJ, USA). Gating of the targeted CD44^+^ and CD24^+^ population was based on the control cells, incubated with isotype control mouse IgG2a-FITC and rat IgG2a-PE.

### 4.6. Immuno-Phenotyping for Expression of Breast Cancer Stem Cell Intracellular Marker ALDH1A1 via Flow Cytometry

Anti-Aldehyde dehydrogenase1A1-APC (Anti-ALDH1A1-APC) flow cytometry was conducted to study the stemness of the MDA subpopulations. The anti-ALDH1A1 (Cell Signaling Technology, Danvers, MS, USA) antibody was first conjugated using the lightning-link APC antibody labelling kit (Innova Biosciences Ltd., Cambridge, UK) to obtain the anti-ALDH1A1-APC antibody. The APC-conjugated anti-ALDH1A1 was prepared at a concentration of 0.01 µg/µL, mixed in PBS. LL-modifier and LL-APC were first added to anti-ALDH1A1 and incubated at 4 °C overnight. LL-quencher was then added and incubated with anti-ALDH1A1-APC at 4 °C for 30 min in the dark according to the manufacturer’s manual and prior usage. Harvested cells were pelleted and fixed using a 4% paraformaldehyde solution (Thermo Fisher Scientific, Arendalsvägen, Göteborg, Sweden) at 4 °C for 10 min. The fixed cells were washed twice in cold PBS and permeabilized using 100 μL of cold 0.25% Triton-X-100 (Sigma-Aldrich, St. Louis, MO, USA) with PBS for 10 min. The permeabilized cells were rinsed twice with cold PBS (incubated for 5 min each time) before being stained with a 1:200 dilution of anti-ALDH1A1-APC in PBS at 4 °C for 1 h in the dark. The cells were washed with PBS to remove unbound dyes. Stained cell pellets were resuspended in 500 µL of fresh PBS, then subjected to a NovoCyte flow cytometer and analyzed using NovoExpress Software (ACEA Biosciences, San Diego, CA, USA).

### 4.7. Immunofluorescence Microscopy Detection of Breast Cancer Markers CD44/CD24 and ALDH1A1

Both pMDA and sMDA were seeded at a cell density of 8000 cells/well in a flat-bottom 96-well plate (BD, Franklin Lakes, NJ, USA) and incubated at 37 °C in 5% CO_2_ for 24 h to achieve 70% confluency. These seeded cells were treated with 56 µM of cisplatin for 48 h. Before the controls and treated pMDA and sMDA were fixed, the medium was carefully removed from wells and cells were rinsed twice with PBS to remove the residue from the medium. Cells in each well were fixed using 50 µL of 4% paraformaldehyde solution (Thermo Fisher Scientific, Arendalsvägen, Göteborg, Sweden) at room temperature for 10 min. Fixed cells were then washed twice using PBS at room temperature and blocked with blocking solution, 1% bovine serum albumin (BSA) (Sigma-Aldrich, St. Louis, MO, USA) in PBS for 1 h at room temperature. Cells were double stained with two antibodies, anti-CD24-FITC (Biolegend, San Diego, CA, USA) and anti-CD44-Alexa-Fluor 647 (Biolegend, San Diego, CA, USA) with a 1:40 dilution of antibodies in PBS at 4 °C for 1 h in the dark, allowing the conjugation of antibodies to the surface markers on the cells. These immunofluorescent samples for ALDH1A1 were fixed similarly, as described above for CD44 and CD24 surface markers but without the blocking step. Fixed cells were permeabilized with 50 μL of cold 0.25% Triton-X-100 with PBS (Sigma-Aldrich, St. Louis, MO, USA) for 10 min. The permeabilized cells were rinsed twice with cold PBS (incubated for 5 min each time) before being stained with a 1:200 dilution of anti-ALDH1A1-APC in PBS, at 4 °C for 1 h in the dark. Both CD24/CD44 and ALDH1A1 stained cells were rinsed twice with PBS (incubated for 5 min each time) before being counterstained with 0.5 µg/mL of Hoescht solution (Sigma-Aldrich, St. Louis, MO, USA) at room temperature for 10 min. The counterstained cells were rinsed once with PBS for 10 min and 100 µL of fresh PBS was added into wells prior to imaging. CD24 and CD44-stained cells were imaged via FITC, Alexa-Fluor 647 and Hoeschst filters, whereas ALDH1A1-stained cells were imaged via APC and Hoeschst filters using a Zeiss microscope (Carl Zeiss AG, Oberkochen, Germany). The captured images were further processed and exported via Zeiss Zen microscope software (Carl Zeiss AG, Oberkochen, Germany).

### 4.8. RealTime-Glo™ MT Cell Viability Assay

The cell viability of pMDA and sMDA treated by anti-cancer drugs including cisplatin, doxorubicin and 5-fluorouracil (5FU) (ranging from 0 µM to 200 µM and at 4 µM and 500 µM, respectively) was measured using the RealTime-Glo™ MT Cell Viability Assay (Promega, Madison, WN, USA). Both MDA subpopulations were seeded at their optimum seeding densities in white 384-well plate (SPL Life Sciences, Pocheon-si, Korea) to achieve 70% confluency after 24 h of incubation at 37 °C in 5% CO_2_. To each well, 60 μL of 1 X RealTime-Glo™ reagent was added just before the addition of the drug (0 h) at different concentrations. Luminescence readings were taken at 0, 24, 48 and 72 h after the addition of the drug. The temperature of each plate was equilibrated to 37 °C using the Varioskan Flash microplate reader (Thermo Fisher Scientific, Arendalsvägen, Göteborg, Sweden) during the reading of plates. The IC_50_ value (the concentration of drugs that reduced cell viability by 50% compared to control cells) of each drug was determined based on the graph of cell viability (%) vs. drug concentration generated through the formula shown in below, to evaluate the cytotoxicity.

$$\mathrm{Percentage}\;\mathrm{of}\;\mathrm{cell}\;\mathrm{viability}\;\left(\%\right)=\frac{\;\mathrm{OD}\;\mathrm{of}\;\mathrm{treated}\;\mathrm{sample}\;\mathrm{at}\;50\%\;\mathrm{cell}\;\mathrm{viability}}{\mathrm{optical}\;\mathrm{density}\;\left(\mathrm{OD}\right)\;\mathrm{of}\;\mathrm{control}}\times 100\%$$

### 4.9. FITC-Annexin V/PI Staining Apoptosis Assay

Harvested cells were washed twice with PBS and resuspended in 1 × binding buffer provided in the FITC Annexin V Apoptosis Detection Kit I (BD, Franklin Lakes, NJ, USA) in which 100 µL of solution, consisting of 1 × 105 cells, was stained by FITC-Annexin V and PI as described in the manufacturer’s manual for the FITC Annexin V Apoptosis Detection Kit I (BD, Franklin Lakes, NJ, USA). The cells were incubated at room temperature for 15 min in the dark. For the identification of apoptotic cells, stained cells were placed in a BD Accuri C6 flow cytometer and analyzed using BD Accuri C6 software (BD, Franklin Lakes, NJ, USA).

### 4.10. Cell Cycle Analysis

Harvested cells were washed with buffer solution (BD Biosciences, San Jose, CA, USA) and subjected to cell cycle analysis using a CycletestTM Plus DNA Reagent Kit (BD Biosciences, San Jose, CA, USA) according to the manufacturer’s protocols. The washed cell pellets were successively resuspended and incubated in solution A (trypsin), solution B (trypsin inhibitor and RNAse A) and solution C (PI) for 10 min, respectively. Stained cells in mixed solution were placed in a NovoCyte flow cytometer and analyzed using NovoExpress Software (ACEA Biosciences, San Diego, CA, USA).

### 4.11. Mammosphere Formation Efficiency Test

A mammosphere formation efficiency test was used to investigate the self-renewability of each subpopulation [33] Harvested cells were seeded into U-bottom 96-well plates (BD, Franklin Lakes, NJ, USA) at a cell density of 200 cells per well and sustained in DMEM/F12 (Sigma-Aldrich, St. Louis, MO, USA) supplemented with 20 ng/µL of epidermal growth factor (EGF) (Peprotech, Rocky Hill, NJ, USA), 20 ng/µL of basic fibroblast growth factor (bFGF) (Peprotech, Rocky Hill, NJ, USA), 1% B27 (Gibco, Gaithersburg, MD, USA), 10 µg/mL insulin (Thermo Fisher Scientific, Arendalsvägen, Göteborg, Sweden), 0.5 µg/mL hydrocortisone (Nacalai Tesque, city, Japan), 1% Penicillin-Streptomycin solution (Gibco, Gaithersburg, MD USA), 1% L-glutamine (Thermo Fisher Scientific, Arendalsvägen, Göteborg, USA) and 0.4% BSA (Sigma-Aldrich, St. Louis, MO, USA). To study the mammosphere formation efficiency of these MDA subpopulations, the seeded cells were observed for 14 days and the numbers of generated mammospheres were counted. Experiments were performed in three biological replicates, each consisting of one technical replicate. Mammosphere formation efficiency was calculated based on the formula shown below.

$$\mathrm{Mammosphere}\;\mathrm{formation}\;\mathrm{efficiency}\;\left(\%\right)=\frac{\begin{array}{c} \;\mathrm{Number}\;\mathrm{of}\;\mathrm{well}\;\mathrm{with}\;\mathrm{generated}\;\\ \mathrm{mammosphere}\;\mathrm{on}\;\mathrm{day}\;14\; \end{array}}{\begin{array}{c} 48\;\mathrm{mammosphere}\;\\ \mathrm{seeded}\;\mathrm{well}\;\mathrm{on}\;\mathrm{day}\;0\; \end{array}}\times 100\%$$

### 4.12. Quantitative Real-Time PCR Array for Cancer Stem Cell-Correlated Putative Target Genes

Total RNA extraction was conducted using QIAzol (Qiagen, Hilden, Germany) according to the manufacturer’s recommendations. Only RNA samples that achieved good purity (absorbance ratios between 1.8 to 2.0 for A260/A280 and greater than 1.8 for A260/A230) were used in this study. DNA contaminants were removed from 2.5 µg of total RNA prior to cDNA conversion using the RT2 first stand kit (Qiagen, Hilden, Germany). The cDNAs obtained were loaded into an RT2 profiler PCR array for a human CSC panel (Qiagen, Hilden, Germany), followed by the addition of the SYBR Green Mastermix (Qiagen, Hilden, Germany). The cDNA was subjected to a real-time polymerase chain reaction using CFX96 (Bio-Rad, Hercules, CA, USA) to quantify gene expression of the CSC-correlated putative target genes in the PCR array. Experiments were performed in three biological replicates that consisted of one technical replicate of each gene for one RT2 profiler PCR array. Putative target gene expression was analyzed using Bio-Rad CFX Manager (Bio-Rad, Hercules, CA, USA), whereas the dysregulation of genes was analyzed using the RT2 Profiler PCR Array Data Analysis Webportal (Qiagen, Hilden, Germany), normalized using beta-2-microglobulin (B2M), hypoxanthine phosphoribosyltransferase 1 (HPRT1) and ribosomal protein lateral stalk subunit P0 (RPLP0) housekeeping genes.

### 4.13. Gene Ontology Enrichment Pathway Analysis of Dysregulated Putative Target Genes

Enrichment of gene ontology (GO) terms of dysregulated putative target gene profiles were determined through the PANTHER classification system (http://www.pantherdb.org/ (accessed on 12 April 2020) [34]. The PANTHER overrepresentation test, version 15.0, was used (Fisher’s exact test with false discovery rate (FDR) multiple test correction) via comparison with GO database release 2020-02-14. *p*-values < 0.05 were regarded as statistically significant for the analysis.

### 4.14. Statistical Analysis

Data were analyzed and graphs were generated using PRISM v8.0 (Graph pad software) with the analyzed standard deviation (SD) displayed as error bars in the respective generated bar charts. Data acquired from RealTime-Glo™ MT Cell Viability were analyzed using Student’s unpaired *t*-test. Remaining bioassays, including CD24/CD44 and ALDH1A1 expression, Annexin V/PI assays, cell cycle analysis and mammosphere formation efficiency test were analyzed using one-way analysis of variance (one-way ANOVA), followed by multiple comparisons testing with the Tukey correction (comparison of mean differences between groups). All data were expressed as mean ± SD of three independent biological replicates, each with three technical replicates. The results were considered to be statistically significant at a probability level of *p*-value < 0.05.

## 5. Conclusions

The findings of this study suggest the higher tolerance of sorted CSC-like cells from MDA-MB-231 to the therapeutic effect of cisplatin. This conclusion was based on our observations of fewer events of late apoptosis/necrosis, less fragmented DNA, a greater protective mechanism against DNA damage and the greater retention of self-renewability in tsMDA, as compared to parental cells after cisplatin treatment. In agreement with the observed cellular characteristics of tsMDA after cisplatin treatment, further putative target gene expression investigations revealed that underlying mechanisms may contribute to higher cisplatin resistance observed in tsMDA, correlated with the dysregulation of SMO, GSK3β, NOTCH2, DLL1 and WNT1, which were associated with Hh and angiogenesis signaling pathways. These annotated signaling pathways were closely correlated to the regulation of chemoresistance and self-renewability in breast cancer. Significantly, the findings are of potential clinical importance in understanding the development of cisplatin resistance in TNBC in association with CSCs. Further research through knockdown or pharmacological inhibition of the proposed putative targeted genes is required in order to validate the current findings. Confirmation of the putative targeted genes and pathways allow future researchers to improve the efficiency of cisplatin to treat TNBC by suppressing the activity of CSCs.

## Data Availability

The data presented in this study are available in the supplementary materials.

## Supplementary Materials

The following are available online at https://www.mdpi.com/article/10.3390/ph14050391/s1, Table S1: Significantly (at least 2-fold) dysregulated mRNAs common to both tpMDA and tsMDA as compared to their respective controls; Table S2: Significantly (at least 2-fold) dysregulated mRNAs observed exclusively in tpMDA and tsMDA as compared to their respective controls.
