# Supplementary material for: Regulation of Cellular and Cancer Stem Cell-Related Putative Gene Expression of Parental and CD44+CD24− Sorted MDA-MB-231 Cells by Cisplatin
Source: Pharmaceuticals (Basel). 2021 Apr 21;14(5):391. doi: 10.3390/ph14050391 (PMC8143088; doi:10.3390/ph14050391)
Supplement: Supplementary file 1 [file pharmaceuticals-14-00391-s001.zip › pharmaceuticals-1143353-supplementary.pdf]

Supplementary

Table S1: Significantly dysregulated mRNA at least 2-fold commonly in tpMDA and tsMDA as compared to their respective controls

Refseq	Symbol	tpMDA vs pMDA		tsMDA vs tsMDA	
		Regulation	p-value	Regulation	p-value
Hs.591293	ALCAM	-10.3708	0.000241	-9.48	0.003445
Hs.590970	AXL	-3.6584	0.000284	-5.7	0.011933
Hs.712050	CD24	-9.2877	0.021891	-10.31	0.030796
Hs.502328	CD44	-3.366	0.007249	-3.04	0.020179
Hs.24529	CHEK1	-2.1762	0.004843	-2.42	0.013949
Hs.129452	DACH1	-17.2568	0.000305	-6.98	0.031733
Hs.631988	DDR1	-15.1365	0.000301	-5.35	0.010887
Hs.40499	DKK1	-12.8463	0.007066	-2.6	0.005304
Hs.511076	DLL4	2.7376	0.009138	2.85	0.000614
Hs.419815	EGF	-7.6401	0.001131	-9.96	0.008957
Hs.542050	EPCAM	-5.0391	0.003102	-2.35	0.007788
Hs.446352	ERBB2	-5.693	0.000615	-4.8	0.003329
Hs.514038	FLOT2	-4.6928	0.00094	-5.73	0.00489
Hs.155651	FOXA2	-4.2726	0.001713	-7.46	0.011321
Hs.59368	FOXP1	-34.7805	0.000262	-19.01	0.012
Hs.88556	HDAC1	-2.4179	0.011826	-4.29	0.017716
Hs.504609	ID1	-16.7571	0.000002	-19.77	0.000305
Hs.597664	IKBKB	-10.9453	0.000002	-6.47	0.001509
Hs.482077	ITGA2	-36.079	0.000417	-30.6	0.006567
Hs.133397	ITGA6	-36.6162	0.003259	-21.37	0.006281
Hs.224012	JAG1	-24.2317	0.014532	-17.69	0.00359
Hs.656213	JAK2	-12.7849	0.000032	-6.87	0.001862
Hs.631951	MAML1	-3.6431	0.001695	-3.7	0.001578
Hs.89603	MUC1	-30.6805	0.024952	-13.34	0.006396
Hs.202453	MYC	-6.0288	0.010922	-2.57	0.029429
Hs.618430	NFKB1	-5.8197	0.007383	-7.83	0.01179
Hs.495473	NOTCH1	-13.0746	0.041332	-12.9	0.007066
Hs.491582	PLAT	-91.2305	0.029918	-29.21	0.010689
Hs.463059	STAT3	-3.595	0.000945	-4.17	0.003564
Hs.409911	TAZ	-6.6513	0.000617	-7.18	0.013042
Hs.494622	TGFBR1	-5.3059	0.003772	-5.22	0.005948
Hs.503692	YAP1	-8.956	0.011124	-6.04	0.020181
Hs.124503	ZEB1	-8.2047	0.000168	-6.07	0.017362

Table S2: Significantly dysregulated mRNA at least 2-fold exclusively in tpMDA and tsMDA as compared to their respective controls

**tpMDA vs pMDA**			
**Refseq**	**Symbol**	**Regulation**	**p-value**
Hs.374990	CD38	-4.7884	0.017924
Hs.647667	KLF17	-13.3461	0.008453
Hs.306178	MERTK	-28.6492	0.000011
Hs.518438	SOX2	4.3413	0.008621
**tsMDA vs sMDA**			
Hs.404102	ABCB5	2.85	0.000614
Hs.76392	ALDH1A1	2.85	0.000614
Hs.367437	ATM	-4.69	0.000642
Hs.434961	ATXN1	-68.11	0.001344
Hs.473163	BMP7	2.85	0.000614
Hs.374990	CD34	2.31	0.021205
Hs.379912	DLL1	2.85	0.000614
Hs.202672	DNMT1	-4.72	0.042227
Hs.76753	ENG	-2.83	0.043573
Hs.39925	ETFA	-2.35	0.010367
Hs.533683	FGFR2	2.85	0.000614
Hs.445733	GSK3B	-6.32	0.016282
Hs.624	CXCL8	2.39	0.014753
Hs.440955	ITGA4	2.85	0.000614
Hs.643813	ITGB1	-6.59	0.003364
Hs.479754	KIT	2.85	0.000614
Hs.549084	LATS1	-3.59	0.007695
Hs.23616	LIN28B	2.85	0.000614
Hs.712553	MS4A1	2.85	0.000614
Hs.25960	MYCN	2.85	0.000614
Hs.635882	NANOG	-3.72	0.029433
Hs.709191	NOS2	2.33	0.005285
Hs.487360	NOTCH2	-8.57	0.001533
Hs.514412	PECAM1	-9.37	0.030896
Hs.249184	POU5F1	-4.33	0.025781
Hs.614734	PROM1	2.85	0.000614
Hs.654514	PTPRC	2.85	0.000614
Hs.642842	SAV1	-2.31	0.047465
Hs.437846	SMO	2.85	0.000614
Hs.48029	SNAI1	13.03	0.001386
Hs.644697	THY1	2.85	0.000614
Hs.66744	TWIST1	2.85	0.000614
Hs.745035	TWIST2	2.85	0.000614
Hs.249441	WEE1	-2.94	0.033911
Hs.248164	WNT1	2.85	0.000614
Hs.484047	WWC1	-9.73	0.006622
Hs.34871	ZEB2	-3.24	0.029514
